# Sleep Technology Intervention to Target Cardiometabolic Health (STITCH): a randomized controlled study of a behavioral sleep extension intervention compared to an education control to improve sleep duration, blood pressure, and cardiometabolic health among adults with elevated blood pressure/hypertension

**DOI:** 10.1186/s13063-023-07658-6

**Published:** 2023-10-10

**Authors:** Kelly Glazer Baron, Jennifer Duffecy, Sara Simonsen, Adam Bress, Molly B. Conroy, Tom Greene, Chelsea Allen, Sofia Vallejo

**Affiliations:** 1 Division of Public Health, Department of Family and Preventive Medicine, University of Utah, 375 Chipeta Way Suite A, Salt Lake City, UT 84013 USA; 2 Department of Psychiatry, University of Chicago, 912 S Wood, Chicago, IL 60612 USA; 3 College of Nursing, University of Utah, Salt Lake City, USA; 4 Department of Population Health Sciences, University of Utah, Salt Lake City, USA; 5 Department of Internal Medicine, University of Utah, Salt Lake City, USA

**Keywords:** Sleep, Hypertension (HTN), Systolic blood pressure (SBP), Diastolic blood pressure (DBP), Cardiometabolic disease (CMD), Sleep coaching

## Abstract

**Background:**

Short sleep duration, defined as < 7 h sleep on weeknights, affects 40% of the US adult population, contributing to the increased risk for cardiometabolic diseases, decreased safety, and poorer mental health. Despite the prevalence of short sleep duration, few studies have tested interventions to extend sleep duration. The objective of this study is to test the effects of a behavioral sleep extension intervention on sleep duration, blood pressure, and other measures of cardiometabolic health among adults with elevated blood pressure or hypertension.

**Methods:**

This is a single-blind, randomized controlled trial to determine the impact of a behavioral sleep extension intervention on sleep duration and cardiometabolic health among individuals with short sleep duration (< 7 h per night) and elevated blood pressure or hypertension (SBP 120–150 mmHg or DBP 80–90 mmHg). After completing the screening, participants will be randomly assigned to either a sleep coaching (intervention) or health education (control) group. The participants will have weekly contact for either coaching or education for 8 weeks (intervention period) followed by monthly coaching or education for the next 2 months (maintenance period). Participants will complete assessment visits, actigraphy, and 24-h ambulatory blood pressure recording at baseline/screening, 8 weeks, and 6 and 12 months. The primary outcome is sleep duration at 8 weeks, and the secondary outcome is blood pressure at 8 weeks.

**Discussion:**

The results of this study will determine the effects of behavioral sleep extension on sleep and cardiometabolic health among adults with short sleep duration and elevated BP/hypertension. The results will inform the feasibility and efficacy of behavioral sleep extension and provide information needed for future multi-site effectiveness studies.

**Trial registration:**

ClinicalTrials.gov NCT04766424. Registered on 21 February 2021.

## Introduction

### Background and rationale {6a}

According to data from the National Health Interview Survey,70.1 million US adults (29.2%) sleep < 6 h per 24-h period [1], below the consensus recommendation of “at least 7 h” needed for health and performance among adults [2]. Therefore, a large portion of US adults could potentially improve health and quality of life through extending sleep duration. Meta-analyses demonstrate compelling evidence that short sleep duration independently increases cardiometabolic diseases (CMD) including a 20% increase in hypertension, 40% increase in diabetes22, and 0.23% higher HbA1c in individuals who have type 2 diabetes [3], risk ratios comparable to other lifestyle risk factors such as overweight and physical inactivity. Experiment sleep restriction increases in BP among healthy and hypertensive participants [4, 5], as well as increasing inflammation [6], insulin resistance [7, 8], and caloric intake [9]. Taken together, the studies demonstrate biological and behavioral effects of short sleep duration on CMD development and management.

Despite the well-known negative effects of short sleep duration, surprisingly little attention has been paid to the development of sleep extension interventions and testing whether such treatments can improve CMD. Studies have demonstrated short-term sleep extension results in decreased insulin resistance [10, 11] decreased appetite [12] and lower intake of fat, carbohydrates, and sugars [13]. Together, this growing body of research demonstrates short-term sleep extension is feasible and may confer health benefits for CMD risk factors. However, these studies are limited by small sample size, high staff involvement in the intervention (e.g., intensive coaching and monitoring), and short-term follow-up. Therefore, our project moved this important research forward, taking it from proof-of-concept to efficacy studies to research conducted in more generalizable clinical populations [14].

### Objectives {7}

The goal of this proposed study is to conduct a randomized controlled trial to test the efficacy of our behavioral sleep extension intervention on 24-h ambulatory BP (ABP) and other key biological and behavioral CMD risk factors among participants with short sleep duration and elevated BP/hypertension. The intervention will be completed over a 12-month period in 3 phases, intervention (weeks 0–8), maintenance (months 2–6), and follow-up (months 6–12), allowing us to test the short-term and extended effects of our intervention.

- Aim 1: To estimate the magnitude of the acute (8 weeks) and extended (12 months) effects of our behavioral sleep extension intervention compared to a health education control group on sleep duration
- Aim 2: To test the effects of our behavioral sleep extension intervention compared with a health education control group on CMD risk factors including 24-h ABP, BMI, body fat, and CMD risk biomarkers (inflammation, glycemic control, lipids)
- Aim 3: To evaluate the effects of the intervention on key lifestyle behaviors (e.g., diet and physical activity) and patient-reported outcomes (sleep quality, sleepiness quality of life)

### Hypotheses

Compared to the education control group, we predict greater improvements in sleep duration in the intervention group in sleep duration (aim 1), 24-h ABP, and other measures of BP and CMD risk (aim 2) and health behaviors and patient-reported outcomes (aim 3). We predict improvements will persist at the 12-month follow-up, but the effects of the intervention will be attenuated over time.

### Trial design {8}

**Fig. 1:**
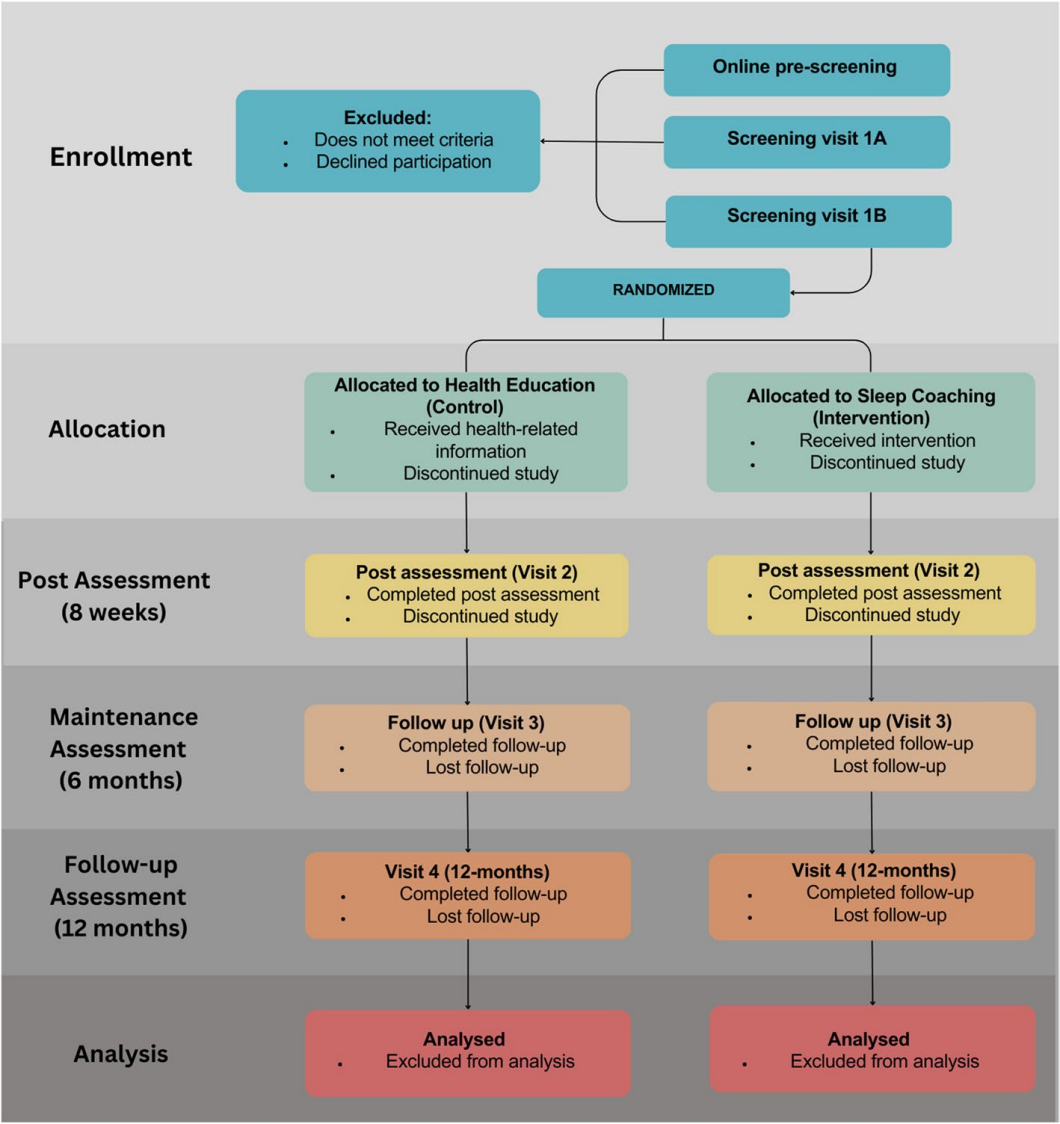
CONSORT-style flow chart for the trial

This is a single-blind, parallel-group, randomized controlled trial with a 1:1 allocation ratio and superiority framework. The study will test a behavioral sleep extension intervention compared to an education control group. The primary endpoint is 8 weeks and the secondary endpoint is 12 months. A CONSORT-style flow chart for the trial is shown in Fig. 1.

## Methods: participants, interventions, and outcomes

### Study setting {9}

This study will recruit participants with elevated BP/stage I hypertension and sleep duration < 7 h from community settings in Salt Lake City, Utah, and the surrounding area.

### Participant timeline {13}

**Fig. 2:**
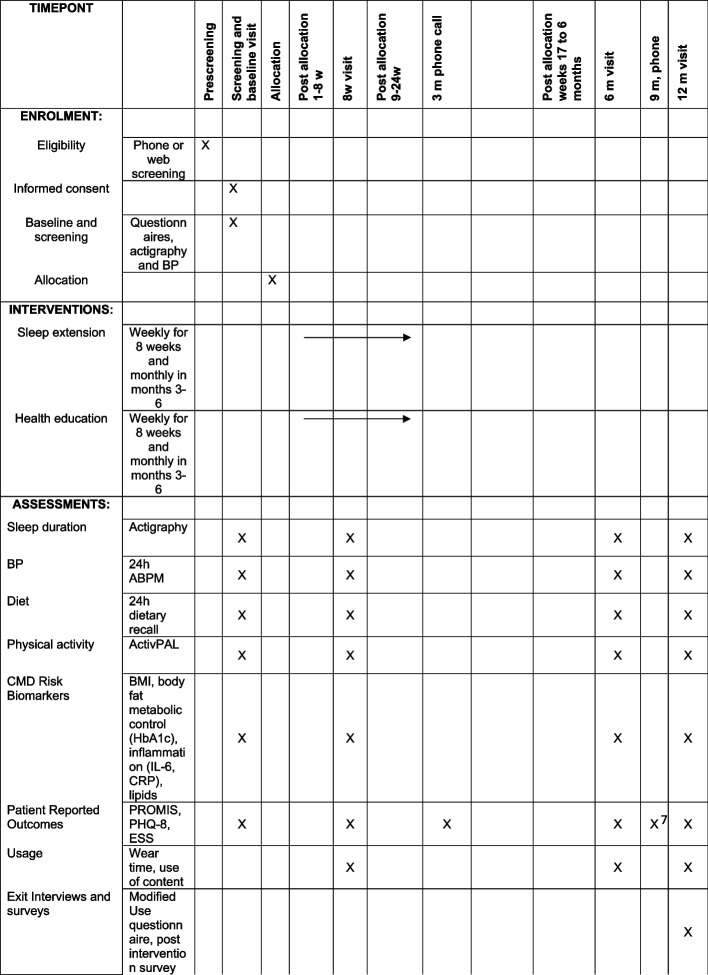
Schedule of time points, study assessments, and interventions

The schedule of time points, study assessments, and interventions is listed in Fig. 2.

After web or telephone screening, participants will complete a baseline/screening visit. Participants will undergo standardized BP screening to determine BP eligibility. The BP screening protocol involves 3 unattended BP recordings taken with an automated BP machine (HEM-907HL, OMRON Healthcare Inc.) after a 5-m quiet resting period and 3 m between measurements. If they meet the study criteria, they will review the consent form with the study staff and complete the informed consent prior to continuing the study visit. Participants will complete self-reported questionnaires and cognitive testing and then will wear the study devices at home over the next 10 days (Apnea link monitor for 1 night, ambulatory blood pressure monitor (OnTrak Ambulatory Blood Pressure Monitor 90,227, Spacelabs Inc.) for 24 h, and actigraphy and physical activity monitor for 10 nights). At the end of the baseline period, participants will return to the lab to download their devices and will have their blood drawn. If eligible for the study, they will be randomized to either the sleep extension intervention group or the education control group. They will receive a study letter and materials in the mail, as well as a call from the study coach informing them of their group assignment. The study interventions have 3 main periods. In the intervention period (study weeks 1–8), participants will receive 8-weekly intervention or control sessions and content. In the maintenance period (months 2–6), participants will receive monthly intervention or control sessions and materials. Finally, in months 6–12, participants will be observed, but there will be no interventions delivered.

### Eligibility criteria {10}

The following are the inclusion criteria:

1. Age 18–65 years.
2. In-office BP readings indicating elevated BP/stage I/II hypertension (SBP of 120–150 mmHg or DBP of 80–90 mmHg). Note that participants taking antihypertensive medications will be permitted if stable dose for > 8 weeks and willing to maintain their dose for the 8-week intervention period.
3. Time in bed < 8 h and habitual sleep duration < 7 h via actigraphy at baseline/screening.
4. Smartphone user.
5. Able to read/write English.

The following are the exclusion criteria:

1. High risk or presence of moderate to severe comorbid sleep disorders (i.e., obstructive sleep apnea, restless legs syndrome, or insomnia) as assessed by the questionnaires and overnight obstructive sleep apnea at the baseline/screening
2. Resistant hypertension, defined as currently taking > 4 antihypertensive medications or 3 medications and standardized in lab BP > 130 mmHg SBP or 80 mmHg DBP at screening
3. History of cognitive or neurological disorders (e.g., dementia, Parkinson’s, multiple sclerosis)
4. Arm circumference > 50 cm
5. Presence of major psychiatric disorders (e.g., schizophrenia, bipolar disorder), alcohol abuse on screening questionnaire (women > 7 drinks per week and more than 4 drinks on one occasion; men > 14 drinks in 1 week and more than 5 on one occasion), drug use on the NIDA-Modified ASSIST (score > 3), and moderate to severe depressive symptoms (PHQ-8 > 9)
6. Unstable or serious medical illness that would interfere with participation (cancer, renal disease on dialysis)
7. Overnight work of more than 1 × per month
8. Use of hypnotic or stimulant medications
9. Situations that would significantly impact the ability to extend sleep, including overnight caregiving responsibility for children under the age 1 and elderly or disabled adults > 1 × per week
10. Inability to read or write in English
11. Pregnancy/desire to become pregnant during the study period
12. Currently following weight loss program, bariatric surgery in the past year, or current use of weight loss medications

### Who will take informed consent? {26a}

Participants will attend a screening visit, and prior to consent, the research staff will take their resting blood pressure using a standardized protocol. Participants who are eligible based on their blood pressure readings will complete informed consent. Consent will be completed by the trained study staff (the research coordinator or research assistant) during an in-person visit to the University of Utah. The informed consent document will be reviewed with participants after a standardized blood pressure reading to determine preliminary eligibility.

### Additional consent provisions for collection and use of participant data and biological specimens {26b}

This study includes blood draws for cardiometabolic risk markers (e.g., lipids, glycated hemoglobin (HbA1c)) as well as Alzheimer’s-related biomarkers (targeted metabolomics, lipidomics) and the genetic risk gene apolipoprotein E4 testing (APOE4). In the consent process, participants will have the opportunity to opt out of blood draws, opt out of genetic testing, and the option to opt out of receiving results of genetic testing.

### Interventions

#### Intervention description {11a}

The behavioral sleep extension intervention consists of a wearable sleep tracker, a weekly educational email, and one weekly phone call from a coach. The maintenance intervention consists of monthly email and phone calls.

**Fig. 3:**
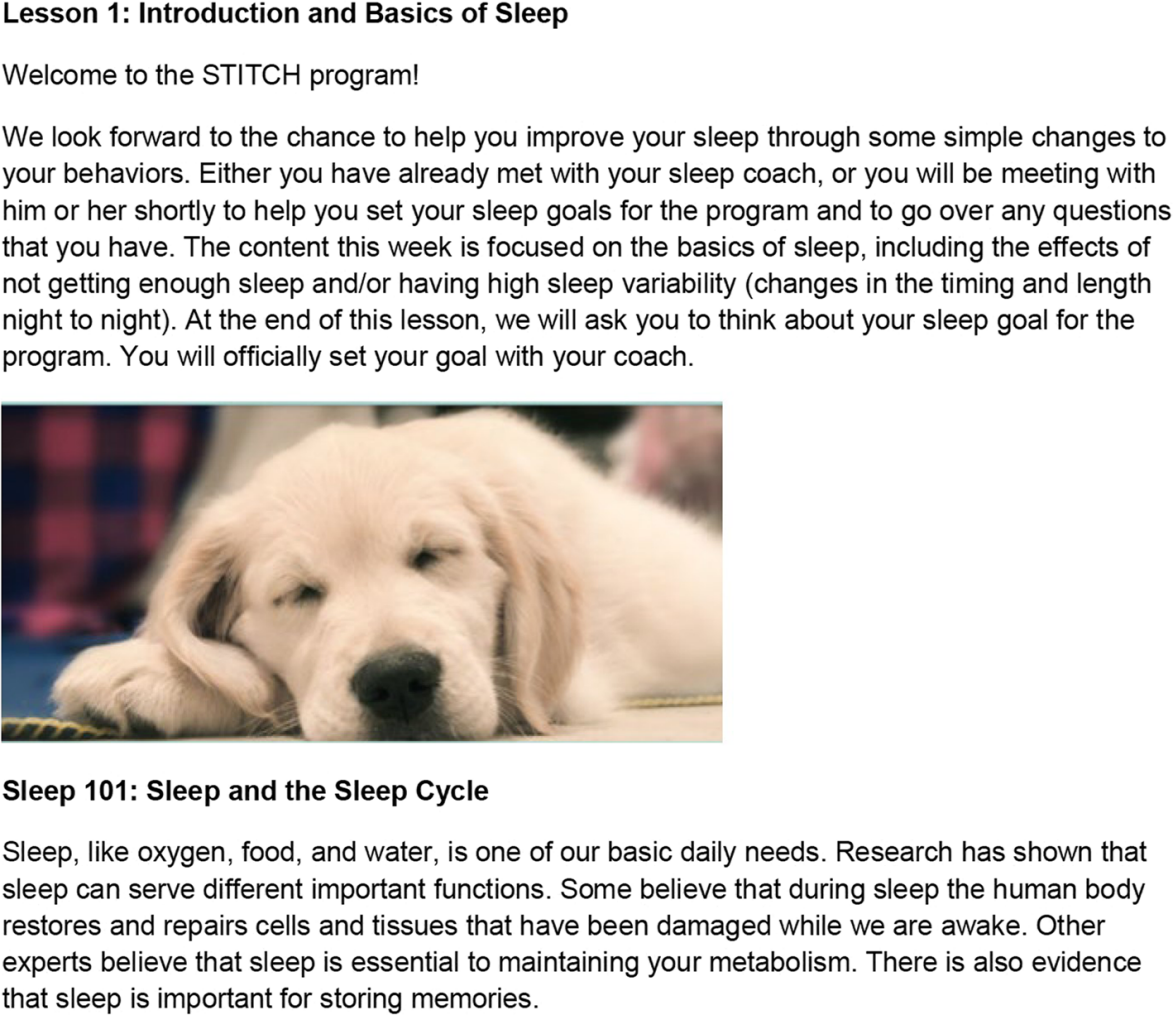
Educational content

1. Self-monitoring (wearable sleep tracker): Participants receive a Fitbit wearable sleep tracker to allow them to monitor their sleep and discuss the results with the coach. Participants and the coach will have access to the sleep tracker data through a dashboard on the Fitabase platform. Participants will be instructed to enter their sleep goals into the sleep tracker app.
2. Education: Participants will receive weekly educational content via email. Topics include the importance of sleep for health (e.g., what is healthy sleep?), regulation of sleep and circadian rhythms, and strategies to avoid bedtime procrastination. The weekly educational content consists of written and video didactic content (e.g., TED talks). Email was selected for the delivery mode to promote engagement, as its use is already integrated into the lives of most people, and it is accessible across platforms (e.g., smartphones, desktops, or tablets). Educational content (Fig. 3) is reinforced in the telephone coaching sessions.
3. Motivational enhancement (telephone coaching): The weekly telephone coaching sessions are used to set goals related to the participant’s sleep, increase commitment to these goals, and problem-solve barriers to achieving the goals. The main behavioral goal is increasing time in bed. The first coaching session will be a 20-min engagement session, which includes introductions, rationale for the program, clarifying the role of the coach, reviewing the participants’ baseline sleep data, setting goals for the program for both sleep and daytime function, and reinforcing the participants’ commitment to change their behavior. In the following coaching sessions, the coach and participant will have a brief (5–10 min weekly) follow-up call to review the progress, boost self-efficacy, problem-solve barriers to progress, and set goals for the following week. During the maintenance period (months 2–6), the calls will be scheduled monthly. In between sessions, the coaches will be available, mostly over email, to troubleshoot any problems with the educational material or sleep tracker. The coaching protocol is based on the principles of Supportive Accountability and uses techniques drawn from cognitive behavioral therapy and motivational interviewing, such as goal setting and developing discrepancies between current and desired behaviors. A list of the intervention and coaching session content is listed in Table 1. Coaches are trained to set collaborative goals, elicit change talk, and include relevant sleep-related research in their sessions (e.g., even an improvement of 20 min can improve your performance).

**Table 1:** Intervention content and coaching

Week	Content	Coaching (min)
1	Intro basics of sleep	20
2	Beat bedtime procrastination	5–10
3	Weekends and challenges	5–10
4	Stress and sleep	5–10
5	The sleep environment	5–10
6	Sleep and the brain	5–10
7	Effects of sleep	5–10
8	Maintaining your gains	5–10

#### Control group description

Participants assigned to the health education control group will be provided with weekly health education emails with content written at < 4th grade level. Participants in this group will also receive brief weekly scripted telephone calls from the coach (< 5 min) to determine if they received the health information and if they had any questions about the materials. Coaches will not provide sleep counseling or goal setting but may clarify terms or concepts. Participants in the control group will be instructed to maintain their current sleep schedule and will not be monitored with sleep diaries, a technique previously reported to produce little change in sleep timing over 4 weeks.

#### Measures of intervention fidelity

All coaching sessions will be recorded for training and fidelity monitoring. All coaches will be trained until they reach a criterion of adherence to the treatment protocol before being assigned study participants. In each quarter, Dr. Duffecy (co-PI) will review approximately 10 sessions quarterly in year 1 and 10 sessions yearly in subsequent years and code them for adherence using previously developed rating scales [15]. Items include aspects of the session content (e.g., eliciting questions or problems about the intervention, setting goals), structure of the session (e.g., sets an agenda), and therapeutic process (e.g., uses open-ended questions, appropriate session pacing). Scores less than 80% will trigger re-training and review of 5 practice sessions for re-approval. Similarly, a random 10% of control sessions will be reviewed using a standardized checklist for the presence of sleep-related content or health advice. If any sleep or health advice is provided, this will trigger re-training.

#### Criteria for discontinuing or modifying allocated interventions {11b}

Participants may be removed from the study if the participant requests withdrawal or the principal investigators consider it necessary to stop the intervention (e.g., significant change that would significantly affect study participations such as pregnancy or a critical injury or major change in health, beginning a shift work job). In this case, the participant would be discontinued from the study, including stopping receiving their emails and coaching sessions. They would receive compensation for study tasks completed up to that point and would not be invited to complete further follow-up assessments. Any information and data collected will be analyzed with the rest of the data.

#### Strategies to improve adherence to interventions {11c}

Retention during the intervention and maintenance periods will be handled by the coaches (e.g., coach will attempt to contact participants via their preferred contact method and reschedule missed sessions when possible). The study staff will also send quarterly study newsletters and birthday and holiday cards to participants throughout the study.

#### Relevant concomitant care permitted or prohibited during the trial {11d)

For the intervention period (weeks 1–8), we will use a constrained usual care model (requesting participants to keep antihypertensive medications constant if possible) to reduce non-study-related variability in the acute intervention phase. For the maintenance and no intervention periods (months 2–12), we will track the medication changes at study assessments but will not require participants to remain at a consistent dose. This plan is based upon current hypertension guidelines that allow for lifestyle interventions for 3–6 months in elevated/stage I hypertension. Both groups will receive written instructions reminding them to follow up as recommended with primary care.

#### Provisions for post-trial care {30}

This trial does not include provisions for post-trial care. If participants inquire about additional sleep interventions after the trial, they will be provided with information about local sleep centers.

### Outcomes {12}

Study outcomes and assessment time points are listed in Fig. 2.

Primary outcome: The primary outcome is a change in sleep duration from baseline to 8 weeks. Sleep duration at baseline and 8 weeks will each be calculated as averages over 10-day periods. Sleep duration will be measured by 8–10 days of actigraphy at baseline/screening, week 8, and 6 and 12 months with the Actiwatch Spectrum device (Philips Respironics Inc.). Devices will be configured using default settings and scored using the Acitware Software (Philips Respironics, Inc.). Actigraphy will be manually scored using a standardized procedure [16].

Main secondary outcome: The main secondary outcome will be change in 24-h SBP from baseline to 8 weeks. This measure will be calculated as an average for the 24-h period at baseline and 8 weeks. BP will be assessed by 24-h ABPM at home using the Spacelabs ambulatory BP monitor (Spacelabs Healthcare, Hertford, UK). Participants will wear the BP monitor at baseline/screening, week 8, and 6 and 12 months. Monitors will be programmed to take readings in random 20-min intervals during the day and 30-min intervals at night.

Other secondary outcomes: Changes from baseline to 6 months and from baseline to 12 months will be measured to evaluate the maintenance of sleep duration changes. In addition, changes in 24-h SBP from baseline to 6 and 12 months will be measured to evaluate the maintenance. We will also measure the change in 24-h diastolic blood pressure (DBP) from baseline to 8 weeks and 6 and 12 months. We will measure diet quality by administering the Automated Self-Administered 24-h Dietary Assessment Tool and calculating the Healthy Eating Index (HEI-2018) [17], a composite measure derived from the 24-h recall measurement that uses food groups and nutrients collected in the diet recall to compute the US dietary guidelines into a single measure. Changes in the HEI reflect the overall change in the healthfulness of diet. We will also compute macronutrients and key food group/diet behaviors (e.g., fast food, fruit/vegetable intake) from the ASA-24 data for use in exploratory analysis.

Objective 24-h physical activity data including sedentary behavior will be collected using the ActivPAL4 activity monitor [18, 19]. The main measure of interest will be minutes of moderate and vigorous physical activity per day (MVPA).

Other measures of cardiometabolic risk will be measured at screening, week 8, and months 6 and 12 including BMI and body fat estimation using a bioimpedance scale (Tanita of America), waist and hip circumference, lipids, and HbA1c. We will also evaluate the effects on nocturnal and diurnal BP and on non-dipping BP status defined by ABPM levels as mean nighttime to daytime SBP/DBP ratio of > 0.90.

Participants will complete measures of patient-reported outcomes at session baseline, week 8, and months 3, 6, 9, and 12, including the PROMIS sleep disturbance, sleep-related impairment and sleepiness [20], and mood [21].

We will assess the intervention acceptability and user engagement to inform our findings and plan for future studies (Fitbit usage, coaching session attendance and duration, and email lesson engagement and use surveys and open-ended questions at the end of the intervention and follow-up periods).

### Participant timeline {13}

**Fig. 4:**
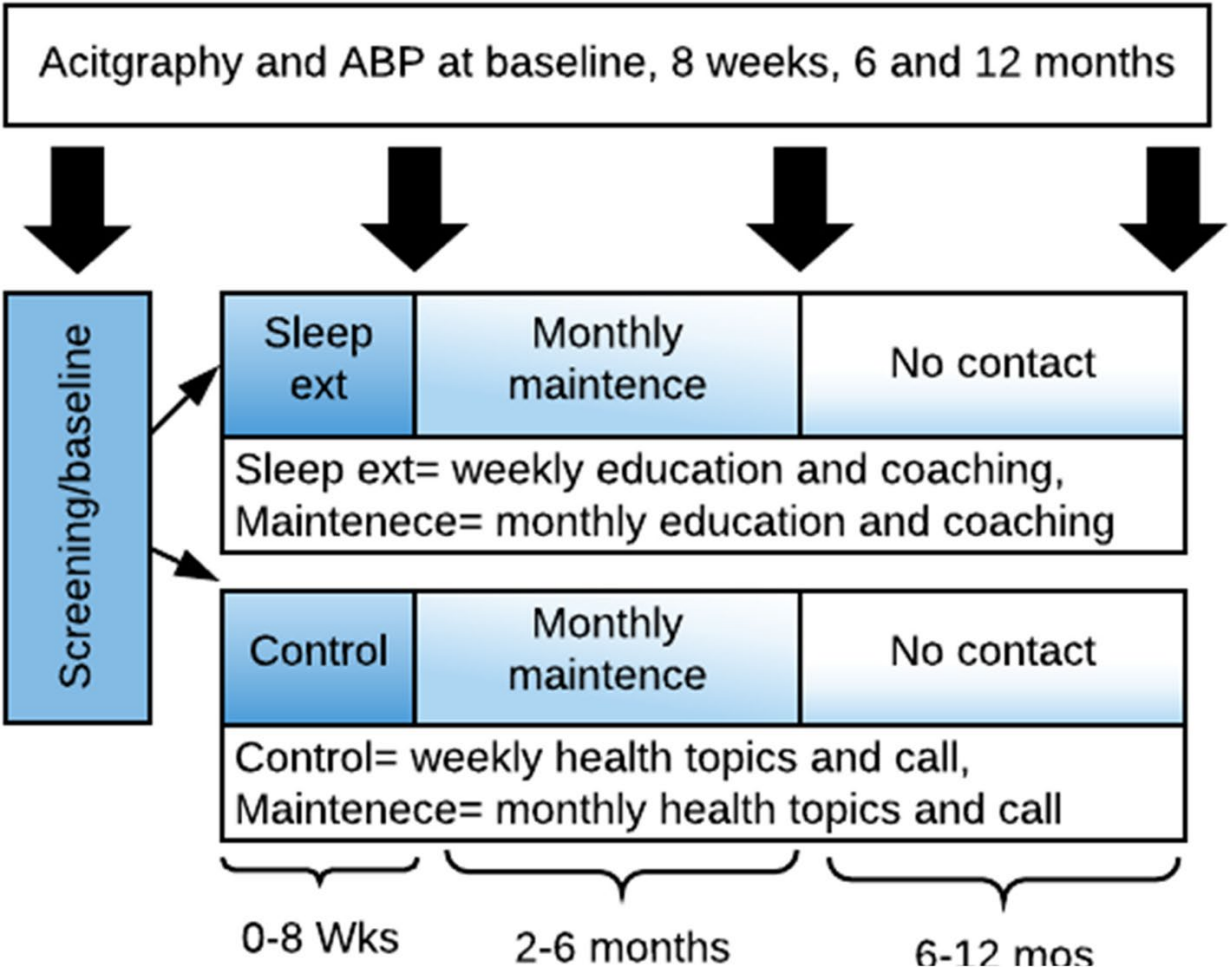
A description of the participant timeline

A description of the participant timeline is listed in Fig. 4.

### Sample size {14}

Our power calculations are based on the assumption that the standard deviation (SD) for change in sleep duration from baseline to 8 weeks will not exceed 37.4 min, which is 10% higher than the SD for change in sleep duration of 34 min reported after 40 days in our previous study [22]. We also assume the Pearson correlation between baseline and follow-up sleep duration will not exceed 0.75, and that the attrition rate will not exceed 20%. Under these assumptions, 120 randomized participants (60 in each of the treatment and control groups) will provide at least 80% power with 2-sided *α* = 0.05 to detect a treatment effect of 20.2 min.

If we also assume that (1) the SD for baseline SBP will be $\le$ 11.8 mmHg, which is 10% higher than the average baseline SD reported in two previous studies [refs]; (2) the Pearson correlation between the baseline and 8-week follow-up SBP values will be $\ge$ 0.70; and (3) the attrition will be $\le$ 20%, and the sample size of 60 patients per treatment group will additionally ensure at least 80% power for the main secondary analysis to detect a treatment effect on the change in SBP of 4.86 mmHg.

### Recruitment {15}

The study will use both active and passive recruitment methods. For active recruitment, patients from the University of Utah Health (UU Health) primary care practices who meet the initial criteria (age, BP) will be sent letters explaining the study and providing information for online screening. The study staff will follow-up letters with a phone call and email to potential participants. We will work with local community leaders and community health workers to enhance the recruitment of diverse study participants, such as providing flyers and information about the study to share with their communities. We will attend local health fairs and conduct BP screening and share study information for interested participants. For passive methods, we will post flyers on the University of Utah campus and nearby areas (e.g., grocery stores and coffee houses) and will post ads on social media (e.g., Facebook). If recruitment slowdowns happen, the team could send more letters, expand to a larger number of clinics, post more frequently on social media, consider in-person recruitment in primary care visits, and attend more health fair events.

### Assignment of interventions: allocation

#### Sequence generation {16a}

Randomization will be performed using random permuted blocks with stratification by sex and antihypertensive medication use.

#### Concealment mechanism {16b}

The randomization allocation will be completed by the biostatistician and loaded into the REDCap system, where it will be concealed from the study investigators and staff.

#### Implementation {16c}

After the screening is completed, the study staff will enter data into a REDCap form including the inclusion/exclusion criteria and stratification information. The interventionist only will have access to the randomization module in REDCap and will complete the randomization and notify the participant of his or her assignment.

### Assignment of interventions: blinding

#### Who will be blinded {17a}

The study staff, the PI, and the co-Is who are not directly responsible for delivering the intervention will be blinded. Participants and the interventionist will be unblinded. The interventionist will not be involved in the collection of outcome measures. The statistician, who creates the randomization table and is completing the analyses will also be unblinded.

#### Procedure for unblinding if needed {17b}

The study has appointed an independent safety monitor, a primary care physician, who is unaffiliated with the study and is available to review the adverse events. He will be unblinded if necessary and will recommend to the study team if a situation arises that unblinding is critical to addressing the situation.

### Data collection and management

#### Plans for assessment and collection of outcomes {18a}

##### Screening measures

Participants will complete self-report measures to evaluate for the presence of elevated depressive symptoms and symptoms of common sleep disorders on the web/phone prescreening. Participants who demonstrate elevated depressive symptoms (score ≥ 15) on the PHQ-8, moderate severity of restless legs (score ≥ 11) on the International Restless Legs Working Group Questionnaire, or severe insomnia on the Insomnia Severity Index [23] and scores ≥ 3 on the STOP questionnaire [24] will be excluded. At the baseline/screening visit (visit 1), participants who are at risk for OSA (STOP = 2) will complete one night with the Apnea Link monitor (Resmed, Inc.) to screen for the presence of obstructive sleep apnea. Participants who have moderate severity or greater OSA (Apnea Hypopnea Index ≥ 15) will be excluded from the study. They will be mailed a notification letter with screening results recommending they discuss the results with their provider.

##### Outcome measures

Primary outcome: Sleep duration at 8 weeks. Sleep will be measured by 8–10 days of actigraphy at baseline/screening, week 8, and 6 and 12 months with the Actiwatch Spectrum device (Philips Respironics Inc.). Devices will be configured using default settings and scored using the Acitware Software (Philips Respironics, Inc.). Actigraphy will be manually scored using a standardized procedure [16]. Participants will be provided with verbal and written instructions. Participants will wear the Actiwatches for 8–10 days in order to maximize the possibility of collecting 7 nights. Data will be considered valid if there are at least 5 nights. After the watches are retrieved, the research staff will use the Actiware software program to calculate the following variables: total sleep time, sleep onset time, sleep offset time, sleep duration, sleep efficiency, sleep latency, wake after sleep onset, and sleep fragmentation index. The staff will be trained on the scoring of actigraphy using a standardized procedure of readings, hands on practice/demonstration, and recorded presentations. Scored of each study file is reviewed by study PI Dr. Baron for scoring quality.

Main secondary outcome: 24-h SBP at 8 weeks. We will use the validated (AAMI criteria) SpaceLabs 90,207 monitor which is generally considered the “gold standard” for ABPM [25, 26]. ABPM for 24 h will be conducted at baseline/screening, 8 weeks, and 6 and 12 months follow-up. At each of the three 24-h ABPM assessments, the proper BP cuff size will be determined by measuring each participant’s arm circumference at the mid-point between the acromion and olecranon. Next, the monitor will be initialized, and the participant will be fitted with a SpaceLabs 90,207 monitor. Participants are instructed to hold their arm still (by their side or with their forearm resting on a desk/table when seated) when a reading is being taken. Each participant will be instructed by the study coordinator to try to keep their arm still and in the neutral position (which will be demonstrated for the participant prior to leaving the visit) from the time that she or he feels the arm cuff begin to inflate until it is fully deflated. The study staff is trained on ABPM procedures by 1:1 demonstration and practice with Dr. Bress, study co-I. After demonstrating the ability to place the ABPM and instruct participants appropriately, the staff members are approved to conduct this study assessment.

Dietary intake will be collected using the ASA-24, an online automated 24-h dietary recall system [27]. The main measure of diet quality will the HEI-2018 [28], a composite measure that uses food groups and nutrients collected in diet recall to compute the US dietary guidelines into a single measure. Changes in the HEI reflect the overall change in the healthfulness of diet. We will also compute macronutrients and key food group/diet behaviors (e.g., fast food, fruit/vegetable intake) from the ASA-24 data for use in exploratory analysis.

Objective 24-h physical activity data including sedentary behavior will be collected using the ActivPAL4 activity monitor [18, 19]. This miniature electronic logger is validated to classify time spent lying, sitting, standing, and intensity of physical activity. The device is placed in a waterproof cover and taped to the participants’ middle anterior aspect of the upper thigh, thus avoiding the need to change accelerometer placement when changing clothes. A picture will be drawn on the waterproof cover to depict the correct orientation of the device. Data will be processed and analyzed using the ActivPAL software according to standardized procedures and best practices [29, 30]. In order to be considered valid data, participants must have 5 days with at least 23 h per day. We are using separate devices for sleep and physical activity because hip/thigh vs. wrist placement is important for measurement of physical activity and sleep [31, 32].

Other measures of cardiometabolic risk will be measured at screening, week 8, and months 6 and 12 including BMI and body fat estimation using a bioimpedance scale (Tanita of America, Arlington Heights, IL), waist and hip circumference, inflammation (IL-6, CRP), lipids, and HbA1c. Training procedures for body measurements include 1:1 training and practice with Dr. Baron and sign off when the staff member can demonstrate following procedures. We will also evaluate the effects on nocturnal and diurnal BP and on non-dipping BP status defined by ABPM levels as mean nighttime to daytime SBP/DBP ratio of > 0.90 [33].

Participants will complete measures of patient-reported outcomes at session baseline, week 8, and months 3, 6, 9, and 12, including the PROMIS sleep disturbance and sleep-related impairment [20], sleepiness [34], and mood [21]. The PROMIS sleep disturbance and sleep-related impairment demonstrate strong internal consistency and have been validated to a mean of 50 and SD of 10 points [20]. The Epworth Sleepiness Scale [35] is a measure commonly used in clinical practice for sleep disorders. The Patient Health Questionnaire, 8-item measure [21] is highly correlated with clinical diagnoses of depression, with scores > 10 associated with elevated depression.

We will assess intervention acceptability and user engagement to inform our findings and plan for future studies (Fitbit usage, coaching session attendance and duration, and email lesson engagement and use surveys and open-ended questions at the end of the intervention and follow-up periods).

Data collection forms are not posted publicly but will be made available upon request.

#### Plans to promote participant retention and complete follow-up {18b}

Retention for the assessment visits will be handled by the study staff. During informed consent, the staff will discuss with the participants the differences between intervention and assessment as well as the impact of dropout on the validity of study results, while also highlighting voluntary participation. For participants who drop out of the intervention (defined as nonattendance of the coaching or health education calls from that point to the end of the intervention period), we will continue to attempt to obtain outcome assessments. We will respond with empathy and attempt to collect primary outcomes if the full assessment is not feasible. Monetary incentives include payment for each study component and a $40 bonus for the completion of all sleep and ABPM time points.

#### Data management {19}

This study will use the secure, web-based Research Electronic Data Capture (REDCap) for data input. Case report forms will be designed to flag out-of-range values. When possible, participants will enter data directly into REDCap. For forms that need to be manually entered (e.g., blood test results, sleep apnea screening), data will be double-checked to verify accuracy. Intervention and control sessions will be stored on Box, a HIPAA-compliant file storage platform. Access to the study data will be limited to the investigators and staff with official roles relevant to the study data.

#### Confidentiality {27}

All investigators and staff will have up-to-date certification on confidentiality and privacy throughout the Collaborative Institutional Training Initiative (CITI) and will have completed additional HIPAA training through The University of Utah. Physical documents will be kept in folders within locked file cabinets in a locked office. Digital documents will be kept on a HIPAA-compliant Box drive or within REDCap. After the trial, data will be kept for 7 years following the last publication of the data. Names and other identifiable information will not be associated with study data. Participants will be assigned a screening ID number and when enrolled into the study, an individual trial ID number.

#### Plans for collection, laboratory evaluation and storage of biological specimens for genetic or molecular analysis in this trial/future use {33}

We will collect and store serum for targeted metabolomics analyses to evaluate whether changes in sleep affect biomarkers related to Alzheimer’s disease risk.

## Statistical methods

### Statistical methods for primary and secondary outcomes {20a}

The primary outcome of the proposed study is the change in the mean sleep duration from baseline to 8 weeks. The study sample size has been determined based on the primary analysis of covariance (ANCOVA) that will compare mean sleep duration at 8 weeks, with statistical adjustment for the baseline sleep duration and the sex and age randomization strata. The proposed ANCOVA approach is recommended for the evaluation of treatment effects on continuous outcomes in randomized trials because it optimally accounts for random imbalances in the outcome at baseline and regression to the mean, thus providing superior statistical power compared to the analyses of change scores or follow-up scores without adjustment for baseline levels [36].

The main secondary outcome for this study is the change in SBP from baseline to 8 weeks. Evaluation of the 24-h SBP outcome at 8 weeks will assure that few patients will have modified their antihypertensive medication regimen over the 8-week interval prior to the primary outcome assessment.

Evaluation of the 24-h SBP outcome at 8 weeks will assure that few patients will have modified their antihypertensive medication regimen over the 8-week interval prior to the primary outcome assessment. Similar ANCOVA models as used with sleep duration will also be used to evaluate the acute effects on this and other numeric secondary and tertiary outcomes.

We will use linear mixed models [37, 38] to analyze changes in sleep duration, BP measures, sleep, and other numeric tertiary outcomes over all follow-up visits, including the 8-week and 24-week assessments. These analyses will evaluate the persistence of treatment effects over the full 24-week follow-up period and will allow us to utilize all data collected (i.e., baseline, 8 weeks, and 24 weeks) while allowing for within-subject variation.

### Interim analyses {21b}

There are no interim analyses planned for this study.

### Methods for additional analyses (e.g., subgroup analyses) {20b}

In addition to our primary analyses, we plan to analyze the results of participants who completed the intervention per protocol, in that they attended all the coaching or health education sessions. We will also conduct exploratory analyses based on clinically significant improvements in sleep and analyze the results among participants in the intervention group that extended their sleep duration by at least 30 min and for those who met criteria at 8 weeks of having sleep duration over 7 h. Finally, we plan to test the impact of obstructive sleep apnea as a moderator of sleep and cardiometabolic outcomes. We will conduct moderator analyses of participants to test the results of the intervention among participants with mild obstructive sleep apnea (5–15 apneas per hour at screening) compared to those with < 5 events per hour.

### Methods in analysis to handle protocol non-adherence and any statistical methods to handle missing data {20c}

We plan to apply multiple imputation to impute missing outcome data, using comprehensive imputation models that include both non-missing measurements from the variables included in the data analysis as well as additional auxiliary variables that are considered to be predictive either of the risk of missingness or the values of the variables whose missing values are being imputed. The multiple imputation approach has been established as superior to alternatives, including complete case analysis or simplified ad hoc single imputation approaches [39, 40].

### Plans to give access to the full protocol, participant-level data, and statistical code {31c}

De-identified data and a codebook will be provided upon written request to the contact PI (Dr. Baron).

### Oversight and monitoring

#### Composition of the coordinating centre and trial steering committee {5d}

This study does not have a coordinating centre or trial steering committee. Dr. Baron and Dr. Duffecy (co-PIs) are jointly responsible for the conduct of the study. Dr. Baron is the contact PI and is responsible for the day-to-day study operations, overseeing the local study staff who are conducting recruiting and data collection activities and responsible for communications with NIH. Dr. Duffecy is responsible for the coaching component of the study, including overseeing the training of the study coach and conducting fidelity assessments of the intervention and control groups. The executive team will meet monthly and is led by Dr. Baron and includes Dr. Duffecy and the co-PIs as well as the project coordinator and staff. The study operations team, including Drs. Baron, Duffecy, the biostatistician (Dr. Allen), study coordinator, and research assistant, will meet weekly to review the study progress and proactively address any potential issues or slowdowns. The data management team (led by Randy Madsen) consists of the University of Utah Biomedical Informatics Core (BMIC), the statistician (Dr. Allen), and co-I/senior statistician, Dr. Greene. This team provides administration and technical staff to support the development of case report forms in REDCap, as well as automated prompt, reminders, randomization modules, and overseeing blinding in the study database. The recruitment and data collection will be conducted by the study coordinator and research assistant. Their day-to-day activities will be supervised by Dr. Baron, who is on site at the research office. There is not a formal stakeholder and public involvement group in this trial.

#### Composition of the data monitoring committee, its role, and reporting structure {21a}

Due to the low-risk nature of the study, there is no data monitoring committee. The study team has appointed an independent safety monitor to review any potential adverse events during the study.

#### Adverse event reporting and harms {22}

Given the non-invasive nature of the intervention, no new adverse events are expected. Adverse events will be assessed at each study visit and between visits if reported during the study period. Adverse events will be reported according to the standard procedures. The team will review such events and rate them as *likely*, *possible*, or *unlikely* in relation to study interventions. Any unexpected serious adverse events or unanticipated problems involving risks to participants that are possibly related to the study will be promptly reported to the PI, safety monitor, IRB, and assigned NINR program officer in accordance with relevant rules and regulations. Investigators will submit yearly progress reports to IRB and NINR summarizing data and safety monitoring activities, including adverse events deemed expected or unrelated to the study and protocol.

#### Frequency and plans for auditing trial conduct {23}

The study investigators will submit an annual report to the study sponsor (National Institute of Nursing) including details of enrollment in the past year, progress on the study aims, and any barriers to completing the study. The study progress will be reviewed by NIH via the yearly progress report. The team will also submit a yearly report to the University of Utah Institutional Review Board (IRB). The team will internally audit the study records yearly throughout the trial period.

#### Plans for communicating important protocol amendments to relevant parties (e.g., trial participants, ethical committees) {25}

Plans for protocol modifications will first be approved by the sponsor (NIH). Then, any modifications will be approved by the IRB and updated in ClinicalTrials.gov. Participants will be notified of the protocol changes, if applicable, at their next study visit, where they will complete an updated informed consent form if necessary. Any protocol deviations will be documented in the protocol deviation form and reported to the IRB.

#### Dissemination plans {31a}

The study team will share updates to the participants approximately quarterly and will send a result summary at the end of the study. The results of the study will be presented at international conferences and publications in peer-reviewed journals. The team will work with their institutions and use social media to highlight the presentations and publications from this study.

## Discussion

This study aims to test the effects of a real-world behavioral sleep extension intervention on sleep and cardiometabolic health among adults with elevated BP/stage I/II hypertension and short sleep duration. Despite the effectiveness of other behavioral sleep medicine treatments, such as cognitive behavioral therapy for insomnia (CBT-I) [41], there are limited interventions to extend sleep among individuals with short sleep duration. Therefore, results of this intervention have the potential to translate behavioral sleep interventions to a sleep problem that is common, associated with mental and physical health and currently not served by existing interventions.

The main feature of our intervention is the use of coaching combined with wearable technology to motivate behavior change. In our previous research, the use of consumer sleep technology was rated as highly desirable to individuals with short sleep duration and, in our intervention development, was rated as the most enjoyable aspect of the study [42, 43]. The addition of the coach is a critical aspect of this treatment. Prior research from our laboratory and others suggests that a wearable device alone is not sufficient to change sleep [44]. The coaching protocol is designed based on the principles of supportive accountability, which includes that the coach is knowledgeable and supportive but also someone who will hold the participant accountable for their behavior [45]. As such, the combination of the wearable device and a supportive person who is viewing and discussing the data with the participant has been successful in our pilot studies in motivating behavior change.

An important limitation to note is that this intervention focuses on extending sleep among individuals who are able to extend their sleep opportunity. We acknowledge that the causes of short sleep duration are multifactorial, and only some aspects are modifiable. Particularly relevant to communities of color, longstanding structural barriers affect sleep contributing to disparities cardiometabolic disease risk [46]. Substantial barriers faced by individuals due to work schedules, child care, and time spent using public transportation are all less modifiable than volitional causes of short sleep duration including bedtime procrastination and leisure activities such as watching TV [47].

In summary, the results of this study will demonstrate the short-term (8 weeks) and longer-term (12 months) effects of behavioral sleep extension. Future research is needed to study in other populations, biopsychosocial contributors to individual differences in who can and cannot extend sleep and also to understand interventions geared toward maintenance of behavior change.

## Trial status

Protocol ver 1.0 (date 06/28/23).

Recruitment began: 3/15/2021.

Anticipated recruitment completion date: 1/1/2024.

## Data Availability

De-identified data and a study codebook will be made available at the end of the study upon request to the contact PI (KB).

## Abbreviations

EMR: Electronic medical records
CMD: Cardiometabolic disease
HTN: Hypertension
SBP: Systolic blood pressure
